# Baseline HOMA IR and Circulating FGF21 Levels Predict NAFLD Improvement in Patients Undergoing a Low Carbohydrate Dietary Intervention for Weight Loss: A Prospective Observational Pilot Study

**DOI:** 10.3390/nu12072141

**Published:** 2020-07-18

**Authors:** Mikiko Watanabe, Renata Risi, Elisabetta Camajani, Savina Contini, Agnese Persichetti, Dario Tuccinardi, Ilaria Ernesti, Stefania Mariani, Carla Lubrano, Alfredo Genco, Giovanni Spera, Lucio Gnessi, Sabrina Basciani

**Affiliations:** 1 Department of Experimental Medicine, Section of Medical Pathophysiology, Food Science and Endocrinology, Sapienza University of Rome, 00161 Rome, Italy; Mikiko.watanabe@uniroma1.it (M.W.); elisabetta.camajani@gmail.com (E.C.); savina.contini@uniroma1.it (S.C.); agnese.persichetti@gmail.com (A.P.); s.mariani@uniroma1.it (S.M.); carla.lubrano@uniroma1.it (C.L.); giannispera@yahoo.com (G.S.); lucio.gnessi@uniroma1.it (L.G.); sabrinabasciani@yahoo.it (S.B.); 2 Department of Endocrinology and Diabetes, University Campus Bio-Medico of Rome, 00128 Rome, Italy; d.tuccinardi@unicampus.it; 3 Department of Surgical Sciences, Surgical Endoscopy Unit, Sapienza University of Rome, 00161 Rome, Italy; ilaria.ernesti@uniroma1.it (I.E.); alfredo.genco@uniroma1.it (A.G.)

**Keywords:** ketogenic diet, very low-calorie ketogenic diet, very low energy diet, very low carbohydrate diet, very low-calorie diet, non-alcoholic fatty liver disease, hepatic steatosis, fibroblast growth factor 21, weight loss, obesity, insulin resistance

## Abstract

Background: Non-alcoholic fatty liver disease (NAFLD) is a major cause of liver disease. Very low-calorie ketogenic diets (VLCKD) represent a feasible treatment as they induce profound weight loss and insulin resistance (IR) improvement. Despite the recognized benefits on NAFLD deriving from pharmacological administration of fibroblast growth factor 21 (FGF21), whose endogenous counterpart is a marker of liver injury, little is known about its physiology in humans. Aim: To identify predictors of NAFLD improvement as reflected by the reduction of the non-invasive screening tool hepatic steatosis index (HSI) in obese patients undergoing a weight loss program. Methods: Sixty-five obese patients underwent a 90-day dietary program consisting of a VLCKD followed by a hypocaloric low carbohydrate diet (LCD). Anthropometric parameters, body composition, and blood and urine chemistry were assessed. Results: Unlike most parameters improving mainly during the VLCKD, the deepest HSI change was observed after the LCD (*p* = 0.02 and *p* < 0.0001, respectively). Baseline HOMA-IR and serum FGF21 were found to be positive (R = 0.414, *p* = 0009) and negative (R = 0.364, *p* = 0.04) independent predictors of HSI reduction, respectively. Conclusions: We suggest that patients with IR and NAFLD derive greater benefit from a VLCKD, and we propose a possible role of human FGF21 in mediating NAFLD amelioration following nutritional manipulation.

## 1. Introduction

Non-alcoholic fatty liver disease (NAFLD) is characterized by hepatic fat accumulation and is not associated with alcohol intake or other known causes of liver disease, and ranges from “benign” fatty liver to significant inflammation and fibrosis. A close mutual relationship links obesity, NAFLD and insulin resistance (IR), leading to the use of the term “metabolic NAFLD”, strongly associated to type 2 diabetes (T2D) [1]. Which the first pathogenetic event between NAFLD and IR is has long been debated. When adipocytes are resistant to the effect of insulin, as in the case of the so-defined IR, lipolysis is increased and the excess plasma free fatty acids (FFA) are taken up by other organs (mainly the liver, but also the heart, muscles, and kidneys) that normally do not store fat, where they constitute ectopic fat depots and they contribute to visceral adipose tissue (VAT) expansion [2]. This is a stronger predictor of increased cardiovascular risk in obese patients compared to Body Mass Index (BMI) [3,4,5,6,7]. On the other hand, NAFLD is a condition that fuels insulin resistance through increased inflammation and lipotoxicity, thus establishing a vicious circle [8].

The obesity pandemic [1,9,10] has led to a skyrocketing increase in the prevalence of many of its complications, some of which are well established, namely T2D, cardiovascular disease, obstructive sleep apnea syndrome (OSAS), and NAFLD [11,12,13,14], and others are currently being further investigated [15,16]. As NAFLD now represents the second cause of liver transplant in the United States, scientists have recently focused on identifying effective means of diagnosis and treatment. The diagnostic gold standard is currently represented by the liver biopsy, but this procedure is rarely performed due to the albeit low risk of serious complications such as bleeding and haemobilia (0.5%). Furthermore, liver biopsy is subject to possible sampling error, and the histopathological staging is operator-dependent [17]. Non-invasive tools include Fibroscan, nuclear magnetic resonance (NMR), and echography. However, none of these is characterized by both good sensitivity and specificity [18], especially in patients with obesity [19]. Therefore, surrogate markers, such as the non-invasive screening tool hepatic steatosis index (HSI), fatty liver index (FLI) and NAFLD liver score (NAFLD-LFS) are still a reasonably reliable and cost-effective method of initial evaluation of fatty liver [20,21]. Although the diagnostic performances of different non-invasive screening methods are difficult to compare as they have been validated against different standards (liver biopsy, ultrasonography, or magnetic resonance spectroscopy) [21], HSI proved to have the highest sensitivity (93%) in ruling in hepatic steatosis at a cut-off <30 and the highest specificity (92%) in ruling out hepatic steatosis at a cut-off > 36, compared to other screening tools [21]. Beside accurate diagnosis and grading from simple hepatic steatosis to steatohepatitis and fibrosis, at present no specific treatment for NAFLD is available, and currently proposed treatments focus on weight loss and insulin resistance improvement [9,22,23,24], with increasing attention being paid in identifying additional safe and effective means of treatment. Among them, ketogenic diets (KD) represent a safe and effective strategy shown to induce beneficial effects on NALFD [25] through a significant and rapid decrease in insulin-resistance and total body and abdominal fat [26,27,28,29,30,31,32,33], but its impact may also be independent of weight change [34]. Moreover, very low-calorie ketogenic diets (VLCKDs) proved effective in reversing T2D through a reduction in triglycerides content and insulin resistance in the liver [33]. Interestingly, the Look AHEAD (Action for Health in Diabetes) study demonstrated that weight loss reduces liver fat in a non-linear fashion, as 5% weight loss reduces NMR-measured intrahepatic triglyceride content by 13%; 11% weight loss reduces it by 52% and 16% by 65%, with the greatest reduction in those undergoing a weight loss above ≥10% [35]. However, it is still unclear whether these thresholds apply to all dietary interventions. Moreover, although it has been reported that diabetic patients with higher baseline insulin levels and shorter disease duration derive greater benefit on glycemic control from a VLCKD [33], close to nothing is known relative to possible baseline predictors of NAFLD improvement, the determination of which can contribute to nutritional therapy personalization for specific patients, based on their metabolic features.

In recent years, considerable attention has been devoted to fibroblast growth factor (FGF21), a protein primarily expressed and secreted by the liver, with autocrine, paracrine, and endocrine effects in multiple target organs [36]. In mice, FGF21 expression is induced in the liver upon acute fasting and a KD, exerting a protective role [37,38]. Interestingly, NAFLD and obesity are related to an increase in serum FGF21, and weight loss leads to its reduction in both mice and men [36]. FGF21 was also recently proven to be an independent predictor and marker of NAFLD in humans [39], and its pharmacological administration has been proposed as an effective treatment to Non-alcoholic steatohepatitis NASH [23]. Despite its re-known pharmacological beneficial effects in condition of ponderal excess both in mice and humans, it has been previously proposed that murine obesity may represent a state of FGF21-resistance [40], although this hypothesis is challenging to translate to humans given the difficulties in the evaluation of FGF21-resistance itself [41]. Different from rodents, serum FGF21 has been shown not to increase upon acute fasting in human subjects [42], whereas KDs resulted in a decrease in FGF21 levels similar to that observed upon weight loss obtained with balanced hypocaloric diets [43,44,45]. It is worthy of note, that both mice and humans experience an increase in circulating FGF21 levels upon hepatotoxic stimuli such as ethanol and fructose consumption [46,47]. Altogether, these concepts explain why defining the physiological actions of endogenous FGF21 is challenging, and whether it is limited to represent a marker of hepatic damage or it also exerts a physiological role in regulating metabolic health in humans is currently a matter of debate.

Considering the importance of identifying an effective treatment for fatty liver and predictors of therapeutic success in NAFLD management, we aimed at evaluating NAFLD amelioration, as reflected by the reduction of the non-invasive screening tool, hepatic steatosis index (HSI), under the cut-off of 36, in obese patients undergoing a 90-day dietary program consisting of a VLCKD followed by gradual carbohydrate and calorie intake increase, and we searched for baseline predictive markers of such improvement among routinely available parameters together with FGF21 serum levels, in order to aid better tailoring of therapeutic interventions for NAFLD.

## 2. Materials and Methods

### 2.1. Study Design and Population

This was a 90-day, single-center, observational prospective study, enrolling patients with a diagnosis of obesity and NAFLD among those accessing the Center of High Specialization for the Care of Obesity, Sapienza University of Rome, Italy. The inclusion criteria were as follows: Age between 18 and 60 years; body mass index (BMI) above 30 kg/m^2^; stable body weight (BW) in the preceding 3 months; positive baseline screening for fatty liver based on HSI > 36. If the patients were diabetic, only treatment with stable doses of metformin were accepted, and those on other medications or uncontrolled T2D were not enrolled. Exclusion criteria included: Hypersensitivity to components used in the protocol products; renal, cardiac, hepatic severe diseases; psychiatric disturbances; type 1 diabetes; previous bariatric surgery interventions; pregnancy or breastfeeding; lack of informed consent.

### 2.2. Dietary Intervention

All patients underwent a 90-day dietary intervention consisting of a first phase, lasting 45 days, during which they consumed a VLCKD with meal replacements (New Penta s.r.l., Cuneo, Italy), followed by a 45-day second phase where a hypocaloric diet with meal replacements and gradual reintroduction of carbohydrates (low-calorie diet, LCD) was followed. Participants received nutritional counseling at baseline and every two weeks up to 3 months, and they were encouraged to exercise for 30 min at least 3 times weekly, although no formal exercise program or incentives were provided.

The VLCKD consisted of the consumption of 5 meal replacements daily and one serving of vegetables with a low glycemic index at lunch and dinner, for a total of approximately 800 kcal/day, with the following macronutrient composition: Carbohydrates 26 g (14%), protein 1–1.5 g/Kg ideal body weight (46%), fat 35 g (40%).

The LCD consisted of the consumption of 6 meal replacements daily and one serving of vegetables with a low glycemic index at lunch and dinner with a gradual reintroduction of carbohydrates (up to 120 g/day), protein intake maintained at 1–1.5 g/Kg ideal body weight, reaching an average calorie intake of 1150 kcal/day by the end of the intervention. 

Meal replacements were composed of whey and vegetable protein derived from soya, green peas or cereals. The Protein Digestibility Corrected Amino Acid Score (PDCAAS), an index describing the protein value in human nutrition, was calculated for the utilized meal replacements to be 0.9 [48].

The dietary fat component mainly came from extra-virgin olive oil, of which the polyunsaturated fatty acids (PUFA) proportion was <10%, the monounsaturated fatty acid (MUFA) was 10–20%, and the saturated fats was <5%. The amount of daily fiber intake was about 27 g/day, mostly deriving from the vegetable servings.

A minimum daily fluid intake of 2 L was recommended, and supplements containing vitamins, minerals and omega-3 fatty acids were provided in accordance with international recommendations relative to dietary interventions composed of meal replacements [49]. The calculated potential renal acid load of the dietary intervention was approximately −6448 mEq/day taking into account the protein containing meal replacements, the recommended vegetable servings, and the provided supplements [50].

### 2.3. Anthropometric Assessment

Anthropometric parameters were measured at baseline and every two weeks thereafter. Body weight was measured using a balance-beam scale (Seca GmbH & Co, Hamburg, Germany). Height was rounded to the closest 0.5 cm. BMI was calculated as weight in kilograms divided by squared height in meters (kg/m^2^). Waist circumference was measured midway between the lower rib and the iliac crest, hip circumference at the level of the widest circumference over the great trochanters to the closest 1.0 cm. Systolic and diastolic blood pressure were measured using an automated digital device.

### 2.4. Body Composition Assessment

Body composition was measured through dual-energy-X-ray absorptiometry (DXA) (Hologic 4500, Bedford, MA, USA) at baseline and at the end of both dietary steps, as previously reported [51]. The abdominal visceral adipose tissue (VAT) cross-sectional area (cm^2^) measurement on the whole-body scan was semiautomated, with the Hologic-developed software locating the outer and inner margins of the abdominal wall on both sides of the DXA image in a 5 cm high region of interest (ROI), the bottom edge, 1 cm above the iliac crest. Total fat mass within the abdominal wall was automatically measured, and the amount of subcutaneous fat between the skin line and the outer abdominal wall was subtracted from the total fat area to obtain the VAT area. Estimated VAT mass (g) was then automatically calculated by the software, based on the area and fat weight [52].

### 2.5. Blood and Urine Chemistry

Full blood count, electrolytes, glucose, insulin, HbA1C, lipid profile (triglycerides, total, HDL and LDL cholesterol), total protein, C-reactive protein (CRP) and erythrocyte sedimentation rate (ESR), plasma creatinine, blood urea nitrogen (BUN), alanine transferase (ALT), aspartate transaminase (AST), uric acid and estimated glomerular filtration rate (eGFR) were determined at baseline and at the end of both dietary steps. The hepatic steatosis index (HSI) was calculated according to Lee et al., 2010 [20]. FGF21 serum levels were measured after an overnight fast using a commercial ELISA assay kit (R&D Systems, Inc., Minneapolis, MN, USA). Insulin resistance was determined through HOMA-IR calculation [53]. Semi-quantitative determination of acetoacetic acid was measured in the first morning urine at baseline and every other week until the end of the study (Ketur-Test, Accu-Chek, Roche Diagnostics, Rome, Italy).

### 2.6. Data Management and Statistical Methods

Statistical analysis was carried out with the statistical package SPSS 25.0 (SPSS, Inc., Chicago, IL, USA). Variables were tested for normality of distribution using the Shapiro-Wilk test. Data are expressed as mean values ± SD (normally distributed variables) and as median values and range (non-normally distributed variables). Variables were log-transformed when non-normally distributed. Comparisons of different time-points were evaluated using mixed-effect analysis, and Tukey’s multiple comparisons tests were used as appropriate. Stepwise regression modeling was used to determine predictors of percentile changes of HSI at different time points, after checking linear regression assumptions. The R-squared coefficient was used to describe the goodness of fit of the regressions. An α error of 0.05 was considered the threshold for statistical significance. The mean ± SD HSI we observed in the population with obesity accessing our clinical center was 43 ± 16. Assuming a power of 0.80 and alpha of 0.05, 55 patients were considered appropriate to highlight a clinically relevant reduction of 15% in the HSI value, leading to reach below the cut-off of 36. Foreseeing a dropout rate of approximately 20%, 65 patients were enrolled.

### 2.7. Ethical Aspects

The study protocol was approved by the Ethical Committee of Sapienza University of Rome (rif. 5475, date of approval 24-10-2019), conducted in accordance with the Declaration of Helsinki and the Good Clinical Practice. All patients were informed about the possible risks and benefits of the proposed interventions and provided written consent.

## 3. Results

### 3.1. Clinical Features

65 participants (23 M, 42 F) were enrolled, with a mean age of 51 ± 11.2 years, whose baseline characteristics, together with changes over time, are reported in Table 1. Briefly, 51 of 65 patients had IR, 14 of 65 patients were diagnosed with T2D and were on stable doses of metformin. Twenty-two patients were taking antihypertensive agents, 11 were dyslipidemic and were being treated with statins, 26 patients were diagnosed with metabolic syndrome (MS) according to NCEP ATP III diagnostic criteria [54]. By the end of the study, 8 patients reduced the doses or stopped metformin, 12 reduced or stopped the antihypertensive therapy; 5 discontinued the lipid-lowering therapy. Before the end of the study, 20 patients dropped-out: 3 during the VLCKD phase, and 17 in the subsequent LCD phase. No significant adverse event was recorded.

### 3.2. Change in Body Mass and Composition

**Figure 1:**
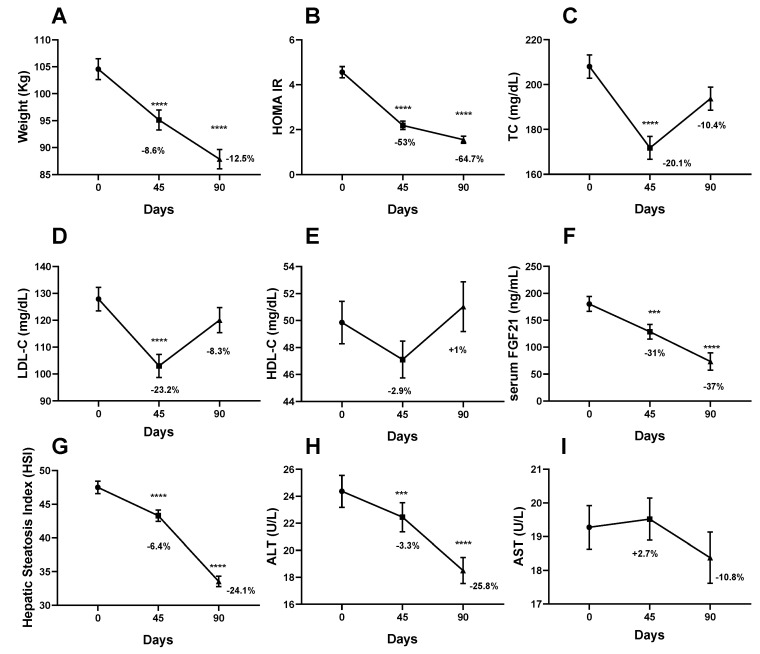
Effect of dietary intervention involving a first VLCKD phase (from T0 to T45) and a second LCD phase (from T45 to T90) on (**A**) weight, (**B**) HOMA-IR, (**C**) TC, (**D**) LDL-C, (**E**) HDL-C, (**F**) FGF21 serum level, (**G**) HSI, (**H**) ALT, (**I**) AST. Data show percentile changes of the variable from T0 to T45 and from T0 to T90. Asterisk (*) denotes statistically significant changes from T0 to T45 and from T0 to T90 at the 0.0001 (***), <0.0001 (****) level. Abbreviations: HOMA-IR, homeostasis model assessment; FGF21, fibroblast growth factor 21; HSI, hepatic steatosis index; AST, aspartate transaminase; ALT, alanine transferase; TC, total cholesterol; LDL-C, LDL cholesterol; HDL-C, HDL cholesterol.

At the end of the study, body weight, BMI and other anthropometric parameters were improved, with major changes occurring during the VLCKD-phase. The average weight loss was −8.6% ± 2.5 after the first step and −12.5% ± 3.7 at the end of the study (Table 1; Figure 1A). Moreover, body composition underwent an overall improvement, with a significant decrease in total fat and VAT along the study. A small but statistically significant decrease in lean mass was also observed, consistent with previous studies [29]. However, the percentage of lean body mass relatively increased, together with a reduction in the percentage of fat along the study (Table 1).

### 3.3. Change in Biochemical Parameters

Both glucose (fasting insulin and glucose, HOMA IR, HbA1c%) and lipid metabolism profiles (total cholesterol, LDL cholesterol, triglycerides) were significantly improved by the end of the study, with the first phase characterized by the greatest change (Table 1, Figure 1B–E). Conversely, creatinine and BUN, as well as inflammatory parameters, did not undergo any significant change along the study period (Table 1). Urinary acetoacetic acid, reflecting ketosis, increased significantly from baseline to the end of VLCKD, and returned to zero by the end of the LCD-program (data not shown).

Baseline circulating FGF21 was 180.1 ± 89.9 ng/mL, with no difference between M and F, T2D and IR, MS and non-MS, dyslipidemic and non-dyslipidemic, hypertensive and non-hypertensive patients (data not shown). Serum FGF21 was positively correlated with diastolic blood pressure (*p* = 0.012), fasting insulin (*p* = 0.004), HOMA-IR (*p* = 0.006), and ALT (*p* = 0.02), confirming its association with conditions of metabolic disruption, whereas no correlation was found with anthropometric and adiposity markers (Table 2). FGF21 serum level decreased significantly from baseline to the end of the study (*p* = 0.0001). Post-hoc analysis confirmed a significant decrease during the VLCKD step (*p* = 0.02); a decreasing trend during the LCD step was also observed (*p* = 0.07) (Table 1, Figure 1F).

### 3.4. NAFLD Improvement and Its Predictors

HSI significantly decreased throughout the study (*p* < 0.0001) suggesting a major reduction in liver fat content, with a smaller drop during the VLCKD phase compared to the following LCD ((*p* = 0.02 and *p* < 0.0001, respectively) Table 1, Figure 1G)). After the VLCKD step, HSI decreased by −6.4 ± 5.7%, whereas a −24.1 ± 7% decrease was observed at the end of the LCD step compared to baseline (Figure 1). Interestingly, liver function tests followed a similar pattern, with both ALT and AST being significantly reduced only after the LCD-phase (*p* < 0.0001, Figure 1H; *p* = 0.008, Figure 1I, respectively). At baseline, all patients had an HSI over 36, which is strongly suggestive for NAFLD [20]. After the VLCKD step, 93.5% still had an HSI over 36. After the LCD step only 20% (9/45) of those completing the study had an HSI over 36, with 57.8% (26/45) having one between 30 and 36, and 20% (9/45) having a value below 30, a cut off under which NAFLD is ruled out with a sensitivity of 93.1% [20].

A stepwise multiple regression analysis was performed in order to elucidate the independent predictors of the percentile changes of HSI from baseline to the end of the VLCKD step and from baseline to the end of the study. Following the identification of possible independent predictors based on Pearson correlations (variables showing a significant association with HSI percentile change (*p* value ≤ 0.05), we included baseline HOMA-IR, FGF21, and VAT as independent variables. HOMA-IR was the only predictor of HSI percentage change throughout the VLCKD, step (*p* = 0009, R = 0.414; Table 3A), whereas baseline serum FGF21 concentration was the only predictor of the HSI change recorded at the end of the study (*p* = 0.04, R = −0.364; Table 3B).

## 4. Discussion

We herein confirm that a multistep VLCKD-LCD dietary approach characterized by meal replacements is safe and effective in reducing body fat and weight, with an improvement in both NAFLD and IR markers [27,33,55]. Our results are in line with those reporting a correlation between circulating FGF21 and metabolic disruption in human subjects. As expected, the VLCKD phase induced a more pronounced reduction of the HOMA-IR, total cholesterol and triglycerides, likely driven by a rapid and significant weight and fat loss, compared to the LCD phase. In addition, many patients reduced or stopped taking antihypertensives, lipid- and glucose-lowering drugs throughout the study as necessary, further confirming the magnitude of the metabolic improvement obtained. The difference in carbohydrate content could have played a role, but the study design did not allow for further clarification. As reported by Rosenbaum et al. [44], circulating FGF21 decreased upon a ketogenic dietary intervention, confirming its lack of involvement in ketosis effects in humans as opposed to murine models [38].

Interestingly, although liver function tests and HSI decreased throughout the entire study, the deepest change was observed in the second, LCD-phase, unlike other biochemical and anthropometric outcomes. This finding, on the one hand supports the hypothesis that VLCKDs exert beneficial effects on NAFLD [25] and, on the other hand confirms the non-linear weight loss-induced improvement of NAFLD previously described [35]. In fact, it has been demonstrated that the greatest benefit of NAFLD obtained from dietary interventions occurs when at least 10% weight reduction is achieved. Although slightly improved after the first phase of the study where the average weight loss was approximately 9%, none of the patients had an HSI value ruling out NAFLD. On the contrary, by the end of the LCD phase, when the average weight loss was 12%, 80% had an HSI incompatible with NAFLD. The pathophysiological mechanism underlying the delay in NALFD improvement compared to other metabolic parameters has never been investigated. It seems reasonable to speculate that the resolution of liver injury itself involves a mechanism slower to set up compared to the improvement of other metabolic parameters. To the best of our knowledge, there are no studies evaluating the rapidity of NAFLD improvement compared to other biochemical variables after nutritional interventions, although some studies evaluating the effects of multi-step dietary regimens including a ketogenic phase collaterally reported a later improvement of transaminases [30,56].

When searching for predictors of fatty liver improvement, we found that baseline HOMA-IR was an independent predictor of greater HSI reduction during the VLCKD step, suggesting that subjects with higher insulin resistance may benefit from a VLCKD intervention in terms of NAFLD improvement, even before a 10% weight loss. This result may be explained by the extremely low carbohydrate content in VLCKDs, which in turn decreases both fasting and postprandial insulin levels with a subsequent possible reduction of ectopic fat.

We also showed that baseline circulating FGF21 was a negative predictor of liver fat reduction throughout the entire study length. Although FGF21-resistance has only been hypothesized in humans, in analogy with the concept of insulin resistance, we could infer that individuals with lower serum FGF21 might be more sensitive to its hepatoprotective action achieving a more pronounced improvement in liver fat accumulation upon a therapeutic intervention. These considerations suggest that human FGF21 could exert an active role in NAFLD improvement, especially in those individuals whose metabolic disruption is not an “FGF21-resistant state”. Circulating FGF21 may therefore not only represent an index of improved liver metabolic status, but it could also predict the therapeutic success of diets in terms of NAFLD amelioration.

Our study has some limitations that should be acknowledged. First, this was an observational, exploratory study, and the absence of a control group prevented any comparison with a balanced isocaloric diet, with no definitive conclusion possibly being drawn regarding the impact of the specific macronutrient composition of the VLCKD on study outcomes. Second, a significant number of patients dropped out before the end of the study, mostly during the LCD phase. As no significant adverse event was reported by patients, we attributed the drop-out to poor compliance with the strict dietary program, especially in the LCD phase when hunger may increase [57,58]. The observed drop-out led to a disparity in the number of patients in the three time points (*n* = 65 at T0, *n* = 63 at T45, *n* = 45 at T90), with consequent limitations in the statistic test’s power. Third, NAFLD was only assessed through a surrogate, non-invasive marker, that despite being cost-effective, is subject to lower sensitivity and specificity compared to more sophisticated methods. Fourth, FGF21-resistance was only hypothesized, and no specific investigation relative to this was carried out. Of note, no validated method of diagnosis of FGF21-resistance is available to date. Fifth, ketosis was only indirectly assessed through semi-quantitative determination of urinary acetoacetate, and no serum b-OH-butyrate was measured. Sixth, patients’ compliance to the prescribed physical activity was unmonitored, and this may represent a bias for the interpretation of our results. Finally, the sample size was relatively small, although power calculation was performed in order to detect a significant liver fat reduction in the study population.

## 5. Conclusions

In conclusion, our preliminary findings suggest that individuals with IR derive greater benefit from a VLCKD as opposed to those with less IR in terms of NAFLD amelioration, hinting that such dietary intervention should be highly recommended to these patients. We also propose a role of endogenous FGF21 in mediating NAFLD improvement following a nutritional manipulation. However, further studies evaluating the link between FGF21 and NAFLD are needed to investigate the timing of liver improvement in response to different dietary regimens and the direct role of FGF21 and FGF21-resistance in ensuring this improvement, possibly through the use of more reliable methods of NAFLD diagnosis and monitoring such as histopathology evaluation.

## Figures and Tables

**Table 1:** Participants characteristics at baseline (T0), after the very low calorie ketogenic diet (VLCKD)-phase (T45) and the low calorie diet (LCD)-phase (T90). Variables with normal distribution are expressed as mean ± SD, those with non-normal distribution as median (interquartile range). Abbreviations: BMI, body mass index; WC, waist circumference; HC, hip circumference; BP, blood pressure; HOMA-IR, homeostasis model assessment-insulin resistance; BUN, blood urea nitrogen; AST, aspartate transaminase; ALT, alanine transferase; HSI, hepatic steatosis index; CRP, C-reactive protein; ESR, erythrocyte sedimentation rate; TC, total cholesterol; LDL-C, LDL cholesterol; HDL-C, HDL cholesterol; TG, triglycerides; VAT, visceral adipose tissue. *p* is from mixed-effects analysis.

	T0 (Mean ± SD) *n* = 65 (a)	T45 (Mean ± SD) *n* = 63 (b)	T90 (Mean ± SD) *n* = 45 (c)	*p*
**Anthropometrics**				
**Weight (Kg)**	104.6 ± 15.3 ^b,c^	95.1 ± 14.1 ^a,c^	87.5 ± 12 ^a,b^	<0.0001
**BMI (Kg/m^2^)**	38.3 ± 6.0 ^b,c^	34.7.8 ± 5.7 ^a,c^	31.3 ± 4.0 ^a,b^	<0.0001
**WC (cm)**	112.8 ± 14 ^b,c^	105.1 ± 17.3 ^a,c^	98.9 ± 9.1 ^a,c^	<0.0001
**HC (cm)**	123.1 ± 10.5 ^b,c^	117.3 ± 10.3 ^a,c^	113 ± 9.8 ^a,b^	<0.0001
**Body Fat (g)**	39824 ± 10492 ^b,c^	34078 ± 10230 ^a,c^	30064 ± 8923 ^a,b^	<0.0001
**Lean Mass (g)**	62680±11608 ^b,c^	61116 ± 12160 ^a,c^	57412 ± 10167 ^a,b^	<0.0001
**Body Fat (%)**	37.3 ± 7.0 ^b,c^	34.7 ± 7.7 ^a,c^	33.3 ± 8.1 ^a,b^	<0.0001
**Lean Mass (%)**	59.8 ± 7.8 ^b,c^	62.9 ± 7.2 ^a,c^	64.1 ± 7.8 ^a,b^	0.0003
**VAT Mass (g)**	862.8 ± 295.9 ^b,c^	781.6 ± 267.5 ^a,c^	689.81 ± 206.9 ^a,b^	<0.0001
**Clinical parameters**				
**Systolic BP (mmHg)**	131 (20) ^b,c^	122.3 ± 13.7 ^a,c^	120 (10) ^a,c^	<0.0001
**Diastolic BP (mmHg)**	80 (15) ^b,c^	70 (16) ^a^	70 (15) ^a^	<0.0001
**Biochemical parameters**				
**FGF21 (ng/mL)**	180.1 ± 88.9 ^b,c^	128.7 ± 87.7 ^a,c^	73.5 ± 55.5 ^a,b^	<0.0001
**HbA1C (%)**	5.6 ± 0.4 ^b,c^	5.3 (0.4) ^a^	5.4 ± 0.27 ^a^	<0.0001
**Glucose (mg/dL**)	98.8 ± 12.6 ^b^	92 (18) ^a^	95.2 ± 8.4	0.001
**Insulin (µUI/mL)**	16.3 (7.8) ^b,c^	8.1 (8.1) ^a,c^	6.4 ± 3 ^a,c^	<0.0001
**HOMA-IR (ng/mL)**	4.5 ± 1.8 ^b,c^	2.2 ± 1.4 ^a,c^	1.5 ± 0.8 ^a,b^	<0.0001
**Creatinine (mg/dL)**	0.8 (1.3)	0.8 (1)	0.8 ± 0.2	ns
**BUN (mg/dL)**	37.9 ± 8.2	39 (65)	40.3 ± 11.4	ns
**AST (U/L)**	19 (7)	19.5 (5) ^c^	17 (6) ^b^	0.008
**ALT (U/L)**	22 (13) ^b,c^	20 (9) ^a,c^	16 (9) ^a,b^	<0.0001
**HSI**	47.5 ± 7.5 ^b,c^	43.3 ± 6.3 ^a,c^	33.5 ± 4.6 ^a,b^	<0.0001
**TC (mg/dL)**	208-1 ± 42.0 ^b^	171.8 ± 38.2 ^a,c^	193.7 ± 32.3 ^b^	<0.0001
**LDL-C (mg/dL)**	127.9 ± 35.3 ^b^	103.0 ± 32.2 ^a,c^	120.1 ± 29.5 ^b^	<0.0001
**HDL-C (mg/dL)**	49.9 ± 12.5	48.7 ± 12.8	48 (12)	ns
**TG (mg/dL)**	125.0 (55) ^b,c^	91 (33) ^a^	90 ± 27.7 ^a^	<0.0001
**CRP (µg/dL)**	3300 (5075)	2350 (3750)	3650 (5350)	ns
**ESR (mm/h)**	26 (19)	25.5 (21)	28 (26)	ns

Letters denote the columns with which a statistically significant pairwise comparison exists.

**Table 2:** Bivariate correlations between baseline serum FGF21 concentration and variables under consideration. Abbreviations: BMI, body mass index; WC, waist circumference; HC, hip circumference; BP, blood pressure; HOMA-IR, homeostasis model assessment-insulin resistance; BUN, blood urea nitrogen; AST, aspartate transaminase; ALT, alanine transferase; HSI, hepatic steatosis index; CRP, C-reactive protein; ESR, erythrocyte sedimentation rate; TC, total cholesterol; LDL-C, LDL cholesterol; HDL-C, HDL cholesterol; TG, triglycerides; VAT, visceral adipose tissue.

	Pearson Correlation Coefficient	*p*
**Anthropometric parameters**		
**Age (years)**	0.132	0.406
**Weight (Kg)**	−0.058	0.715
**BMI (Kg/m^2^)**	−0.032	0.842
**WC (cm)**	0.077	0.626
**HC (cm)**	−0.139	0.379
**Total Fat (g)**	−0.153	0.333
**VAT Mass (g)**	0.140	0.382
**Systolic BP (mmHg)**	0.136	0.389
**Diastolic BP (mmHg)**	0.397	**0.009**
**Biochemical parameters**		
**HbA1C%**	0.213	0.192
**Glucose (mg/dL)**	0.123	0.445
**Insulin (µUI/mL)**	0.400	**0.012**
**HOMA-IR (ng/mL)**	0.388	**0.015**
**Creatinine (mg/dL)**	0.106	0.510
**BUN (mg/dL)**	−0.089	0.586
**AST (U/L)**	0.234	0.147
**ALT (U/L)**	0.346	**0.025**
**HSI**	−0.083	0.603
**TC (mg/dL)**	−0.075	0.635
**LDL-C (mg/dL)**	−0.113	0.475
**HDL-C (mg/dL)**	−0.077	0.634
**TG (mg/dL)**	0.054	0.741
**CRP (µg/dL)**	−0.117	0.478
**ESR (mm/h)**	−0.222	0.158

**Table 3:** Multiple regression analysis to assess predictors of percentile changes of hepatic steatosis index (HSI) during the VLCKD phase (**A**), and throughout the entire study period (**B**). Independent variables evaluated: Baseline FGF21, visceral adipose tissue mass (VAT mass), homeostasis model assessment-insulin resistance (HOMA-IR).

**A**	**Independent Variable**	**Standardized β**	**SE**	**Sig.**	**R**	**R Change**
	**Baseline HOMA-IR**	0.414	0.483	0.0009	0.414	0.172

**B**	**Independent Variable**	**Standardized β**	**SE**	**Sig.**	**R**	**R Change**
	**Baseline serum FGF21 level**	−0.364	0.014	0.04	0.364	0.132
